# Correspondence

**Published:** 1887-01

**Authors:** Thomas Skinner

**Affiliations:** London, England


### CORRESPONDENCE.

### "Whom the Cap Fits, Let Him Wear It."

Dear Mr. Editor:—In your November number I observe a paper purporting to be on "Boycotting by the Mongrels," into which my name is dragged quite unnecessarily; and what connection it has with the title of the paper is completely beyond my comprehension, unless it is upon the principle of Bishop Berkeley's famous book on "tar water" as a panacea for every abdominal ailment, while his real object was to disseminate his own peculiar views about the TRINITY.

This is the second time within a few months that my writings have been critically noticed by your correspondent, and it is remarkable that, although I have been intimately acquainted with him since 1874, he has never, before the month of July last, ventured to honor me with a critical notice of any of my numerous writings in our journals. There must be some cause at work to explain this, and as I feel certain that there will be a succession of attacks of the kind and in the same *animus* from your correspondent, in self-defense I think it right and just to explain what I conceive to be the cause of these recent personal attacks upon me. Last springtime your correspondent and I quarreled—not for the first time, *but for the last*, and he knows it. I say no more on this point, as it fully explains the *animus* which at present and for some time past has influenced him; and be it remembered that he does not criticise recent papers of mine—those which he has done me the honor to take notice of are old ones which I am justly proud of. The first was published by you in this journal as far back as last March, and was not critically noticed until the July following, which was after our final difference. The second was a case of leucocythœmia, which I published in the last volume of the *North American Journal of Homœopathy* so far back as August, 1884; so that your correspondent has been taking his own time about pitching into me. I suppose he had been all this time "nursing his wrath to keep it warm," and since last spring his leisure time has been occupied in rummaging through all my writings so that he might find or make some holes in my coat (query? possibly to injure me in the eyes of my transatlantic Hahnemannian brethren). Your correspondent is much mistaken if he thinks that anything that he can write by way of criticism, considering the *animus* of it, will ever tempt me to reply, and I shall give you my reasons.

Your correspondent's simile of the Irishman's challenge to all comers at Donnybrook Fair reminds me very much of himself, "Wull any jintleman thread on the tail ov me coat?" I am afraid that, although not at Donnybrook, but on the free soil of England, I have inadvertently tramped upon this Irishman's coat, when up goes his shillelah and down it came upon my foot. But he missed it that time, and in turn I gave him a "Roland for his Oliver." It was my reply to an "Explanation Wanted" (*H. Ph.*, July, 1886, p. 256), and which you kindly published in your September number, p. 321. It is presumable that the explanation was satisfactory, as it has never been answered, probably because it was unanswerable even by your correspondent, who is nothing if not critical. But this "explanation" was too much for Celtic blood to stand. There was nothing more in THE HOMŒOPATHIC PHYSICIAN of mine that he could tackle, and as I had written a good deal in *The North American*, he pounces upon my case of leucocythœmia, which he ignorantly thinks is the same as anæmia. Up goes his Hibernian weapon, "which never misses fire," and down it comes upon my devoted cranium; but thanks to its thickness, its hardness, and general niggerly properties, for a moment or two I felt stunned, and as the concussion passed off I drew myself up, felt more in the possession of my senses than ever, and the following were my reflections: Why should I reply to an unfriendly attack upon my writings by a man who has the daring to quote from memory the facts of a case of the utmost urgency and difficulty, a case which one meets with, perhaps, only once or twice in a lifetime? Why reply to one who mangles the facts and selects only those which suit his inimical ends? Why bandy words in self-defense with a man who has an unwarrantable conceit in his own opinions and in his ability to spot the *simillimum*, as if no one could do so but himself? Has your critical correspondent *never* failed to find the remedy, the *simillimum?* Why defend myself against a man who accuses me of using a palliative (Chloroform) in *one solitary case* of the most intense suffering, where, on account of the utter impossibility of getting a word out of the patient, I was driven to do what any man with a heart not made of stone would do? Why argue with a man about the use of Chloroform who knows nothing of it and who never used it, who is frightened by it, whilst I have administered gallons of it since 1847, and was brought up to have the most perfect confidence in it by the discoverer of its marvelous anæsthetic properties, Sir James Simpson, Baronet? Why argue with a man who charges me in 1884, after ten years of practice as a pure Hahnemannian homœopath, with being "still in the Egyptian darkness of allopathy"? I call this something very like the height of impudence, especially when every one of your readers must know that with this single exception I have never had recourse to any palliative of an allopathic kind since 1875, and no one knows this better than your correspondent. Is your correspondent aware that when he quotes a case from memory he not only does the writer injustice, but he lays himself open to the suspicion that it may be done with a purpose —a back-door through which he may make his exit when desirable? Why lower myself by mixing with a man who considers his "guineas" might be an inducement to the British Homœopathic Society to desire his membership? Lastly, my last reflection was a stunner, and I expect that it will prove a greater stunner to your carping critic of a correspondent. When I reflected that he, although practicing as an obstetrician, does not know the difference between a virgin *os uteri* of fifty summers and the *os uteri* of a woman at the ninth month of pregnancy—*vide Medical Advance* of May, 1886—I may safely conclude that I feel warranted in future in declining to take the slightest notice of anything which comes from the pen of your quasi-amiable correspondent—at least, until he shows a very different *animus* toward me. I have to apologize for the length of my letter, but as it is the first and the last of the series, I trust that you will give it publicity.

"If caps amongst a crowd are thrown,
The one which fits you, is your own."

I have the honor to remain, dear sir, yours truly,

THOMAS SKINNER, M. D.

LONDON, ENGLAND, November 20th, 1886.

